# Application of Convolutional Neural Network in the Diagnosis of Cavernous Sinus Invasion in Pituitary Adenoma

**DOI:** 10.3389/fonc.2022.835047

**Published:** 2022-04-14

**Authors:** Yi Fang, He Wang, Ming Feng, Hongjie Chen, Wentai Zhang, Liangfeng Wei, Zhijie Pei, Renzhi Wang, Shousen Wang

**Affiliations:** ^1^Department of Neurosurgery, The Fuzong Clinical Medical College of Fujian Medical University, Fuzhou, China; ^2^Department of Neurosurgery, Fuzhou General Hospital, Fuzhou, China; ^3^Department of Neurosurgery, Peking Union Medical College Hospital, Chinese Academy of Medical Sciences and Peking Union Medical College, Beijing, China

**Keywords:** pituitary adenoma, deep learning, magnetic resonance imaging, cavernous sinus invasion, transfer learning

## Abstract

**Objectives:**

Convolutional neural network (CNN) is a deep-learning method for image classification and recognition based on a multi-layer NN. In this study, CNN was used to accurately assess cavernous sinus invasion (CSI) in pituitary adenoma (PA).

**Methods:**

A total of 371 patients with PA were enrolled in the retrospective study. The cohort was divided into the invasive (*n* = 102) and non-invasive groups (*n* = 269) based on surgically confirmed CSI. Images were selected on the T1-enhanced imaging on MR scans. The cohort underwent a fivefold division of randomized datasets for cross-validation. Then, a tenfold augmented dataset (horizontal flip and rotation) of the training set was enrolled in the pre-trained Resnet50 model for transfer learning. The testing set was imported into the trained model for evaluation. Gradient-weighted class activation mapping (Grad-CAM) was used to obtain the occlusion map. The diagnostic values were compared with different dichotomizations of the Knosp grading system (grades 0-1/2-4, 0-2/3a-4, and 0-3a/3b-4).

**Results:**

Based on Knosp grades, 20 cases of grade 0, 107 cases of grade 1, 82 cases of grade 2, 104 cases of grade 3a, 22 cases of grade 3b, and 36 cases of grade 4 were recorded. The CSI rates were 0%, 3.7%, 18.3%, 37.5%, 54.5%, and 88.9%. The predicted accuracies of the three dichotomies were 60%, 74%, and 81%. The area under the receiver operating characteristic (AUC-ROC) of Knosp grade for CSI prediction was 0.84; the cutoff was 2.5 with a Youden value of 0.62. The accuracies of the CNN model ranged from 0.80 to 0.96, with AUC-ROC values ranging from 0.89 to 0.98. The Grad-CAM saliency maps confirmed that the region of interest of the model was around the sellar region.

**Conclusions:**

We constructed a CNN model with a high proficiency at CSI diagnosis. A more accurate CSI identification was achieved with the constructed CNN than the Knosp grading system.

## Introduction

Pituitary adenomas (PAs) are common intracranial tumors. Although considered benign, 25%–55% of PAs present invasive behavior, characterized by the invasion of adjacent structures, including sphenoid sinus, diaphragm sellae, and cavernous sinus (CS) (1). Cavernous sinus invasion (CSI) is a significant risk factor for incomplete resection, and it is responsible for the failure of endocrinological remission and high rate of tumor recurrence (2–4). Hence, CSI is an important concern in clinical practice. Gross total resection is a challenge in PAs with CSI, considering the ease of surgical injury in the trunk and branches of the internal carotid artery (ICA). In addition, invasiveness is an essential biological behavior of aggressive PAs, in which temozolomide is recommended. The European Endocrine Association guidelines emphasize the importance of radiological invasiveness for aggressive PAs (5). Therefore, a radiological assessment for CSI is essential for the diagnosis and management of PAs.

Considering the close relationship of ICAs, the histological specimens of the medial wall of CS are not routinely available. Intraoperative observations are still the gold standard for CSI. Currently, the application of endoscopes in transsphenoidal surgery enables the surgeon to inspect the medial wall of CS directly and clearly and thus make a reliable judgment about CSI. Pre-operative prediction relies on the Knosp grading system. In 1993, the classification was proposed to quantify the CSI pre-operatively (6). However, the full scale only underwent minor modification (dividing grade 3 into 3A and 3B) during the 30 years (7). This grading system could not accurately predict CSI, especially the intermediate grade (grades 2–3) (8, 9). Even false-positive CSI was present in cases with Knosp grade 4 (9, 10). According to the results of a recent meta-analysis, grades 2, 3A, and 3B presented the CSI rates of 30%, 62%, and 81%, respectively (11). Hence, the dichotomization of the scale into invasive and non-invasive PA is inappropriate. Moreover, the poor percentage agreement among raters for the full scale is disputed.

Consequently, a method that can accurately predict CSI and reduce observer bias is highly required. Deep learning is an essential branch of machine learning (ML) and has improved image recognition and classification (12–14). Machine identification and classification can efficiently facilitate imaging assessment, and it can well avoid observer bias. Thus, this study aimed to construct a deep learning model for radiological CSI diagnosis combined with surgical observations.

## Materials and Methods

### Patient Cohort

All data were obtained from patients with PA who underwent transsphenoidal surgery between 2016 and 2020 at two centers, namely, the Fuzhou General Hospital and Peking Union Medical College Hospital. The review boards of the two medical centers approved this study. The requirement for informed consent was waived because of the retrospective nature of the study.

Patients with clear T1-enhanced imaging suitable for analysis, a surgical record of CSI, and a pathological diagnosis of PA were included. Cases with other intracranial tumors, a previous history of surgery or trauma in the sellar region, and artifacts were excluded.

### Knosp Grading System and Surgical Findings

The Knosp grading system was assessed on the coronal magnetic resonance imaging (MRI) scans. Three lines that connect the cross-section of the intracavernous and supracavernous ICAs (e.g., medial tangent, cross-sectional line, and lateral tangent) distinguish four grades of parasellar adenoma extension. Grade 3 could be subclassified because the tumors extended into the superior CS compartment (3A) or inferior CS compartment (3B). Intracavernous ICAs completely encased by a tumor are defined as grade 4 (7). An experienced neurosurgeon and radiologist assessed Knosp grades together to reduce personal observation error, and the maximum grade was recorded.

CSI was diagnosed based on surgical evidence. If the medical wall of CS is vague, and CS structures are visible, or an invasion was directly visible, CSI is confirmed. If the medial wall is smooth and intact after resection, CSI is considered to be absent. A total of 102 patients were classified into the invasive group, and 269 patients were included in the non-invasive group.

### Image Selection

All imaging data were extracted on the coronal T1 contrast-enhanced scans. Images were evaluated by two senior neurosurgeons with over 10 years of experience in the PA diagnosis and management of performed CSI radiological evaluation. Only one image that was evaluated with potential CSI was enrolled in a case with surgically confirmed CSI. Images with the maximum area of the tumor were selected in PAs without CSI. In the cohort, 371 images were subjected to a fivefold division of the randomized dataset for cross-validation.

**Figure 1:**
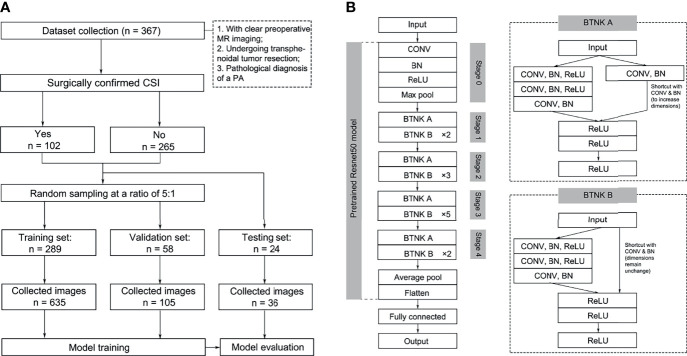
Image selection **(A)** and model structures **(B)**. BN, batch normalization; BTNK, bottleneck; CON, convolution.

The principal investigator from each center assessed and collected images separately. Patient information was filtered and eliminated, and only the acquired images were retained. Finally, the adjudicator screened these images for proofreading and adjudicating to reduce observer bias. The acquisition process is illustrated in **Figure 1**. The imaging parameters of the two centers are shown in the **Supplementary Material**.

### Image Pre-Processing

The image pre-processing procedure is summarized as follows: 1) all images were converted into 256 × 256 square images by using zero padding and image resizing as appropriate, and 2) augmentation procedure was carried out by using the horizontal flip and rotation (within 90°) in the training and validation sets. The augmented dataset was obtained for model training (15).

### Transfer Learning Methods

Convolutional neural networks (CNNs) are among the deep learning models used to recognize and classify images, and they are characterized by multiple layers of feature maps, including convolution (a method in computer vision and signal processing) and pooling (replacing redundant information with representative values) (16). At the end of the network, feature maps are connected to the fully connected layer as the network’s output. The parameters of deep learning models need to be adjusted by backpropagation with respect to the loss function. These models require vast amounts of data for training and millions of parameters for fitting. However, obtaining clinical data is label intensive, especially for rare diseases. Transfer learning could achieve a satisfying model performance with limited samples by using a pre-trained model. The pre-trained models have been trained on a large dataset. The use of a feed-forward approach to adjust partial weights based on input data can rapidly and efficiently train models (17).

The model framework was constructed, and fine-tuning was performed using the pre-trained Resnet50 model. Resnet was proposed in 2015; it is a CNN with a 50-layer network structure consisting of a series of residual modules, and it essentially solves the problem in which multi-layer network models lead to vanishing or exploding gradients, which is conducive to the convergence of network models (18). The Resnet50 model was pre-trained on ImageNet containing 1,000 classes and over 14 million images in the present study. The output of CNN’s fully connected layer was modified into two nodes for final classification to PAs with or without CSI (**Figure 1**, **Supplementary Figure 1**) . Except for the fully connected layer, all weights in Resnet50 were fixed during training. The CNN model was trained with a learning rate of 0.0001 based on the training and validation sets. After constructing the classification model, the testing set was used to evaluate the diagnostic accuracy. The above models were trained and tested using fivefold cross-validation.

### Statistical Analysis

SPSS (version 25) was used for statistical analysis. Categorical variables were expressed as numbers (percentages) and analyzed using the chi-square test. Statistical significance was considered at *p* < 0.05.

Confusion matrices were constructed according to intra-operative diagnosis and pre-operative prediction. The contingency tables of the Knosp grading system were constructed using three dichotomizations (0-1/2-4, 0-2/3a-4, and 0-3a/3b-4). True positives, true negatives, false positives, and false negatives were recorded. The diagnostic index included sensitivity, specificity, a positive likelihood ratio (+LR), a negative likelihood ratio (-LR), precision, and predictive accuracy. The summary of diagnostic odds ratio (DOR), F-score, and area under the receiver operating characteristic (AUC-ROC) were used as the composite index to evaluate the diagnostic performance. DOR is the ratio of +LR to -LR. F-score is the harmonic mean of precision and recall. Gradient-weighted class activation mapping (Grad-CAM) was used to identify the features deduced with the convolutional filter application.

All imaging data-processing and model methods were implemented using Pytorch (version1.8.1, https://pytorch.org) and operated in JupterNotebook (version 6.4.0, https://jupyter.org).

## Result

### Knosp Grading System and Cavernous Sinus Invasion

The cohort of 371 patients included 212 males and 159 females with a mean age of 55.52 ± 11.71 years. A total of 102 (27.5%) patients were surgically confirmed with CSI, and 269 (72.5%) patients were surgically confirmed without CSI (**Table 1**). According to the Knosp classification, 20 cases (5.4%) of grade 0, 107 cases (28.8%) of grade 1, 82 cases (22.1%) of grade 2, 104 cases (28.0%) of grade 3A, 22 cases (5.9%) of grade 3B, and 36 cases (9.7%) of grade 4 were recorded with the invasion rates of 0%, 3.7%, 18.3%, 37.5%, 54.5%, and 88.9%, respectively. A significant difference was found in the CSI at different Knosp grades (*p* < 0.001). By comparison, the reliability for the middle grades was weak.

**Table 1:** Summary of results of Knosp grades.

Knosp Grading System	CSI		Total n (%)	Invasion rate %
	No	Yes		
**Grade 0**	20	0	20 (5.4)	0
**Grade 1**	103	4	107 (28.8)	3.7
**Grade 2**	67	15	82 (22.1)	18.3
**Grade 3a**	65	39	104 (28.0)	37.5
**Grade 3b**	10	12	22 (5.9)	54.5
**Grade 4**	4	32	36 (9.7)	88.9
**Total**	269	102	371	

**Figure 2:**
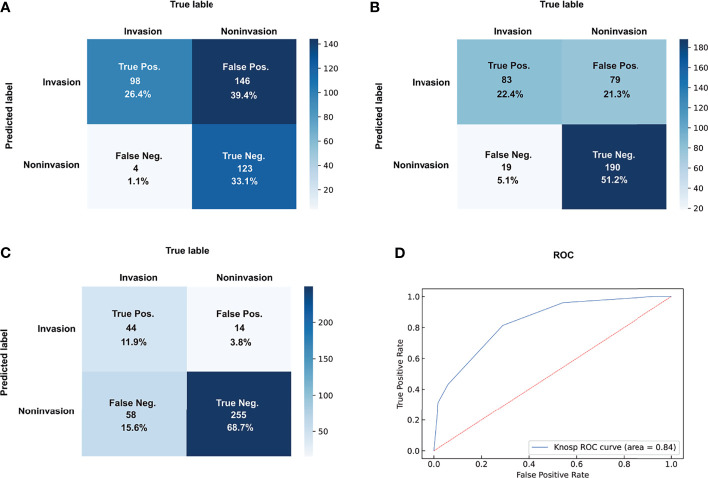
Dichotomizations of grades 2–4 **(A)**, grades 3–4 **(B)**, and grades 3B–4 **(C)** for CSI prediction and ROC of Knosp grades **(D)**. pos., positive; neg., negative.

The details of the diagnostic indicators of the Knosp grading system are summarized in **Table 2** and **Figure 2**. The grades 0–1 presented the high reliability of the cases without CSI, with a sensitivity of 0.96 and -LR of 0.09. However, low reliability was observed for this dichotomization to recognize positive CSI, with poor specificity (0.46), +LR (1.77), precision (0.4), and predictive accuracy (60%). The assessment criterion for CSI (grades 3b–4) in the modified Knosp classification had high reliability among cases with CSI, with a specificity of 0.97 and +LR of 8.29. By contrast, this dichotomization had weak reliability in negative CSI recognition, with poor sensitivity (0.43) and -LR (0.60). The dichotomization of 0-2/3a-4 had an F-score of 0.63, which is higher than the two other dichotomizations. The dichotomization of 0-1/2-4 had a higher DOR value of 20.59, which can be attributed to the low -LR.

**Table 2:** Dichotomization of Knosp grades and the deep learning model for CSI.

Group		TP	FP	TN	FN	Sen.	Spe.	+LR	-LR	DOR	Precision	F-score	Accuracy
**Knosp Grading System**	Grade 0-1/2-4	98	146	123	4	0.96	0.46	1.77	0.09	20.59	0.40	0.57	0.60
	Grade 0-2/3a-4	83	79	190	19	0.81	0.71	2.77	0.26	10.51	0.51	0.63	0.74
	Grade 0-3a/3b-4	44	14	255	58	0.43	0.95	8.29	0.60	13.81	0.76	0.55	0.81
**Deep Learning Model**	Testing Fold 1	17	11	43	4	0.61	0.91	7.13	0.43	16.61	0.61	0.69	0.80
	Testing Fold 2	19	1	53	2	0.95	0.96	26.13	0.05	503.50	0.95	0.93	0.96
	Testing Fold 3	16	7	47	4	0.70	0.92	8.87	0.33	26.86	0.70	0.74	0.85
	Testing Fold 4	17	3	51	3	0.85	0.94	15.3	0.16	96.33	0.85	0.85	0.92
	Testing Fold 5	16	6	47	4	0.73	0.92	9.27	0.30	31.33	0.73	0.76	0.86

DOR, diagnostic odds ratio. FN, false negatives. FP, false positives. -LR, negative likelihood. +LR, positive likelihood ratio. sen., sensitivity; spe., specificity; TN, true negatives. TP, true positives.

Finally, the Knosp grading system had an AUC-ROC value of 0.84, with a maximum Youden index of 0.62 and a cutoff value of 2.5. The overall reliability for the full Knosp grading system was strong in the low (0–1) and high (4) grades.

**Figure 3:**
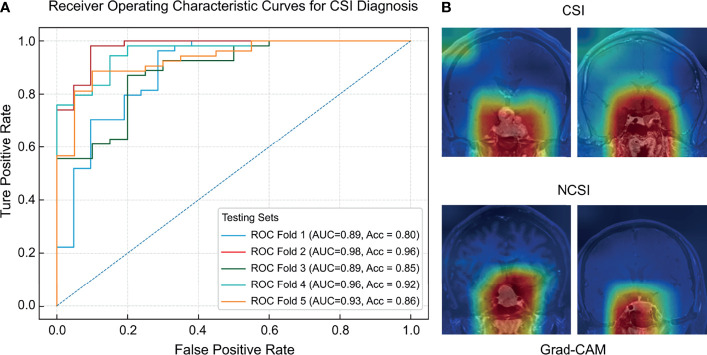
The ROC curves **(A)** and regions of interest **(B)** of the deep learning model for CSI diagnosis.

### Predictive Models for Cavernous Sinus Invasion Recognition

The predictive capabilities of the CNN model were presented after fivefold cross-validation, as shown in **Table 2**. An AUC-ROC value of 0.98 was recorded for the optimal model (**Figure 3**). The model performance was considerably higher than that of the three dichotomizations of the Knosp grading system. The confusion matrixes of the results of the five-fold cross-validation are shown in **Supplementary Figure 2**.

The sensitivity and specificity of the optimal model were 0.95 and 0.96, respectively. The DOR (16.61–503.50) and F-score (0.69–0.93) of the deep learning model were higher than those of the dichotomizations of the Knosp grading system (DOR: 13.81–20.59, F-score: 0.55–0.63). The AUC-ROC value of the model (0.89–0.98) was higher than that of the Knosp grading system. Therefore, the CNN model has higher diagnostic reliability than the Knosp grading system. Grad-CAM saliency maps showed that CSI prediction was made by the attention to the large area around the sellar region, unanimous with the contributing areas in tumor location.

## Discussion

Radiological characteristics are essential for the pre-operative prediction in CSI. Currently, the pre-operative diagnosis of CSI is mainly based on Knosp grading and modified Knosp grading. In the studied cohort, Knosp grades 0–1 and 4 have high accuracy in recognizing positive CSI. The low and high Knosp grades can effectively facilitate CSI evaluation. However, intermediate grades (grades 2–3) presented weak reliability, especially grades 2–3A (11). PAs in intermediate grades are common in clinical practice. In the series, the PAs are of grades 2–3 in 208 (55.4%) and grades 2–3A in 186 (49.5%). Therefore, the PAs are inappropriate to be classified into invasive and non-invasive groups according to the dichotomizations of the Knosp grading system. The weak diagnostic reliability of the intermediate grades leads to a gradual decrease in the dependence of neurologists and radiologists on the radiological diagnosis of CSI based on Knosp grades.

ML enables the use of existing imaging facilities and common sequences to capture information per pixel through computer algorithms and accurately classify diseases, thus reducing the hardware and time costs required to optimize image quality. Deep learning models have been applied for the examination of intracranial tumor in processes such as the diagnosis and classification of PAs (19–21). The latest study on CSI identification with the use of the support vector machine model included 194 PA image data with Knosp grades 2–3 (1). The AUC-ROC values of the training and testing sets were 0.85 and 0.83, respectively. Results show that the ML model could determine cases with CSI in the intermediate Knosp grades and confirm the feasibility of an ML model for PA classification. However, radiomics rely on manual delineation and segmentation, which involve rater bias. Furthermore, the cumbersome operation of manual segmentation is not conducive to clinical promotion.

Our study describes a CNN model based on T1 contrast-enhanced MRI sequences to achieve high-accuracy CSI prediction. With the development of algorithms and computer modules, a transfer learning method that uses a pre-trained model can reduce the training burden (22, 23). Our model is based on the Resnet50 model pre-trained on a massive number of extract images. Deep learning has some applications in PAs (23). In the present study, the diagnostic values of the CNN model for CSI are more reliable than the Knosp grading system. Moreover, the ML model can effectively avoid interrater and intrarater bias.

The accurate identification of PA invasiveness can facilitate the formulation of diagnosis and management and the prognostic evaluation. CSI is an essential indicator for whether chemotherapy or post-operative adjuvant chemotherapy should be considered (24). Therefore, the pre-operative assessment of tumor invasiveness is related to therapy strategies and communication with patients before therapy. In addition, PAs with CSI are not easily carried out for inexperienced endoscopists to achieve gross total resection. In cases with a pre-operative diagnosis of CSI, experienced senior neurosurgeons are recommended to perform tumor resection for a high resection rate. Hence, the accurate radiological diagnosis of CSI can facilitate effective communication and therapy strategy adjustment. The present study focused on a CNN model that can identify CSI more accurately than the conventional imaging grading system. This model can also compensate for the low manual reading efficiency and interrater and intrarater bias.

### Strength and Limitations

The CSI was evaluated based on the T1-enhanced MRI sequences. The image samples are readily available without the additional particular sequences or expensive advanced equipment. Sample collection is convenient for multicenter applications and studies. The ML model could increase radiological diagnostic accuracy and unify the pre-operative radiological diagnosis to reduce rater bias. After model classification, neurologists and radiologists can focus on the misidentified cases to determine more radiographic findings of PAs with negative and positive CSI. Furthermore, conventional imaging grading systems have limitations in terms of updating rates, thus decreasing the reliability with the innovation of medical technologies. By contrast, the ML model has a high learning ability, which is characterized by fast updating and adjustment. With the expansion of the image library, the model can be trained to improve the diagnostic accuracy and generalization ability.

However, this study has some limitations. For example, although the diagnostic accuracy of the model is higher than that of the Knosp grading system, the diagnostic accuracy and generalization ability should be further improved. In addition, the retrospective nature of the study leads to some limitations. For example, in the absence of complete surgical videos and detailed surgical records of the CSI location and area, image selection relied on a senior neurosurgeon to improve the reliability of whether CSI exists in the selected image. Therefore, the observer error still exists. These factors might influence the predictive performance of the CNN model in the study. Therefore, prospective studies with a reliable record of CSI are required for the ML model training. Nevertheless, our study can demonstrate the feasibility of the CNN model for pre-operative CSI diagnosis.

## Conclusions

CSI can be accurately identified by using the conventional MR sequences as input into the pre-trained CNN model. We constructed a CNN model that can more reliably determine the CSI than the Knosp grading system. Moreover, the rater bias of the Knosp scale could be reduced effectively.

## Data Availability Statement

The raw data supporting the conclusions of this article will be made available by the authors, without undue reservation.

## Ethics Statement

All investigations conformed to the principles outlined in the Declaration of Helsinki and were performed with permission by the responsible Ethics Committee of the Institutional Review Board of Fuzhou General Hospital and Peking Union Medical College Hospital. The requirement for informed consent was waived because of the retrospective nature of the study.

## Author Contributions

All authors made a substantial contribution to the research design, acquisition, analysis, or interpretation of data; revised the manuscript critically; and approved the final version. The authors thank SW, RW, and MF for reviewing the manuscript. LW carried out statistical analyses. Medical writing and editorial support were provided by HW and WZ.

## Funding

The Fujian Medical University Sailing Fund Project (grant no. 2019QH2043).

## Conflict of Interest

The authors declare that the research was conducted in the absence of any commercial or financial relationships that could be construed as a potential conflict of interest.

## Publisher’s Note

All claims expressed in this article are solely those of the authors and do not necessarily represent those of their affiliated organizations, or those of the publisher, the editors and the reviewers. Any product that may be evaluated in this article, or claim that may be made by its manufacturer, is not guaranteed or endorsed by the publisher.

## Supplementary Material

Supplementary Figure 1 The structure of ResNet50 model.

Supplementary Figure 2 Confusion matrix of testing sets.
