# Supplementary figures and images for: Application of Convolutional Neural Network in the Diagnosis of Cavernous Sinus Invasion in Pituitary Adenoma
Source: Front Oncol. 2022 Apr 14;12:835047. doi: 10.3389/fonc.2022.835047 (PMC9047893; doi:10.3389/fonc.2022.835047)

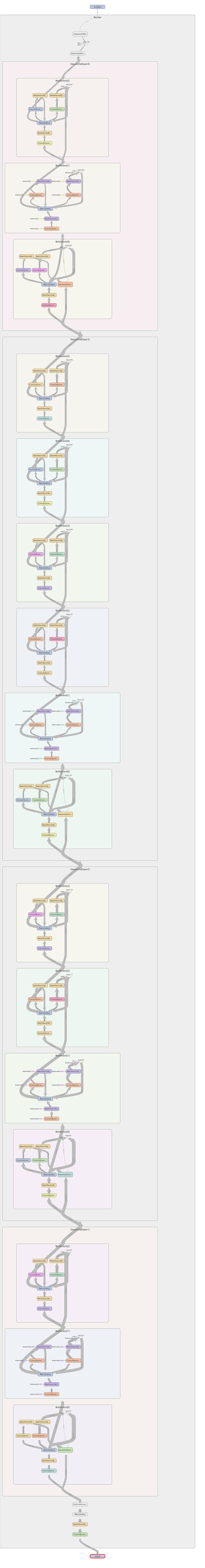

Supplement: Supplementary Figure 1 — The structure of ResNet50 model. [file Image_1.tif]

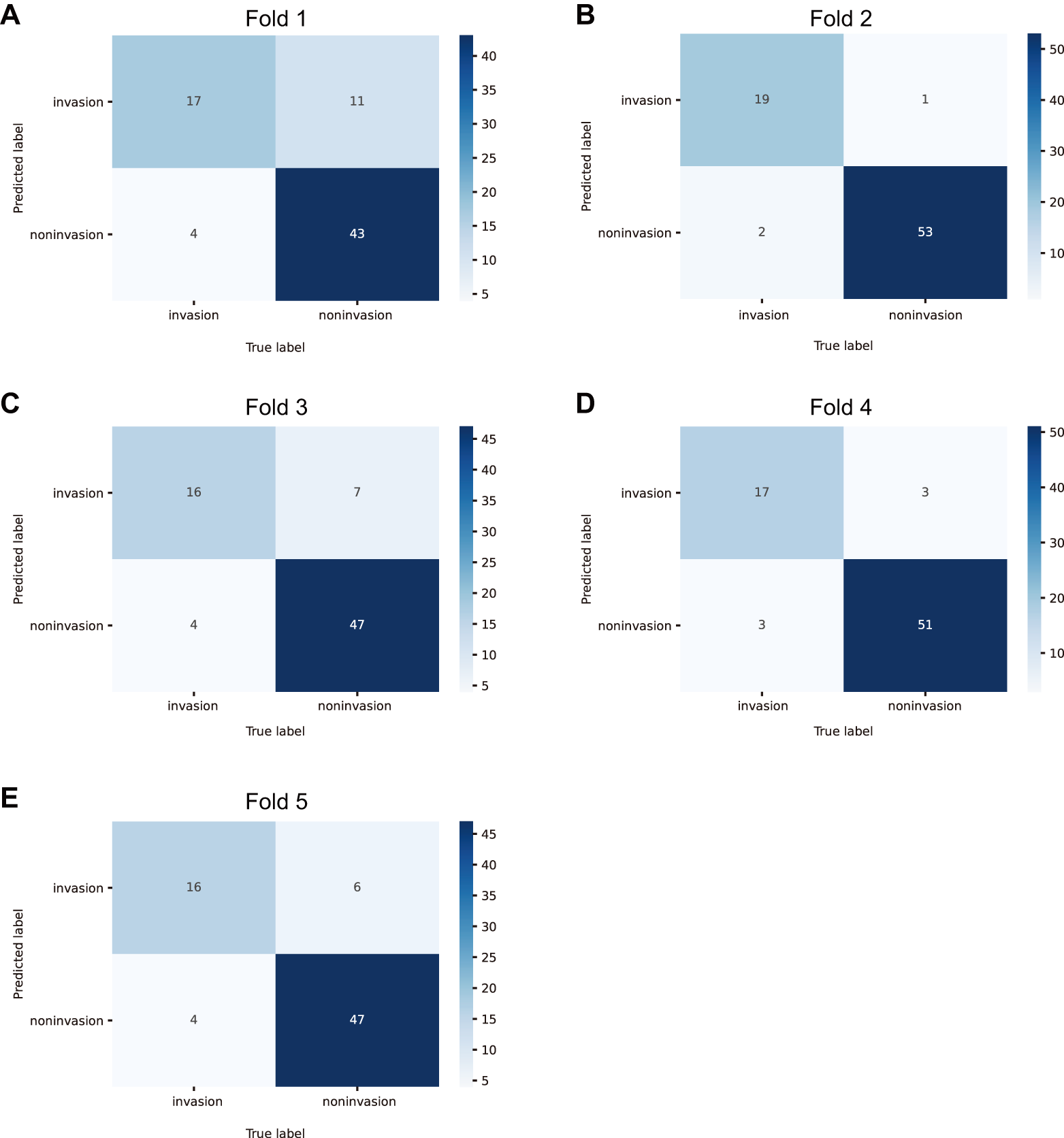

Supplement: Supplementary Figure 2 — Confusion matrix of testing sets. [file Image_2.tif]
